# Ribosomal RNA of *Hyacinthus orientalis* L. female gametophyte cells before and after fertilization

**DOI:** 10.1007/s00425-012-1618-x

**Published:** 2012-03-08

**Authors:** Katarzyna Niedojadło, Szymon Pięciński, Dariusz Jan Smoliński, Elżbieta Bednarska-Kozakiewicz

**Affiliations:** Department of Cell Biology, Institute of General and Molecular Biology, Nicolaus Copernicus University, Gagarina 9, 87-100 Toruń, Poland

**Keywords:** Egg cell, Embryo sac, Fertilization, Nucleolus, RNA polymerase I, Zygote

## Abstract

The nucleolar activity of *Hyacinthus orientalis* L. embryo sac cells was investigated. The distributions of nascent pre-rRNA (ITS1), 26S rRNA and of the 5S rRNA and U3 snoRNA were determined using fluorescence in situ hybridization (FISH). Our results indicated the different rRNA metabolism of the *H. orientalis* female gametophyte cells before and after fertilization. In the target cells for the male gamete, i.e., the egg cell and the central cell whose activity is silenced in the mature embryo sac (Pięciński et al. in Sex Plant Reprod 21:247–257, 2008; Niedojadło et al. in Planta doi:10.1007/s00425-012-1599-9, 2011), rRNA metabolism is directed at the accumulation of rRNPs in the cytoplasm and immature transcripts in the nucleolus. In both cells, fertilization initiates the maturation of the maternal pre-rRNA and the expression of zygotic rDNA. The resumption of rRNA transcription observed in the hyacinth zygote indicates that in plants, there is a different mechanism for the regulation of RNA Pol I activity than in animals. In synergids and antipodal cells, which have somatic functions, the nucleolar activity is correlated with the metabolic activity of these cells and changes in successive stages of embryo sac development.

**Electronic supplementary material:**

The online version of this article (doi:10.1007/s00425-012-1618-x) contains supplementary material, which is available to authorized users.

## Introduction

The female gametophyte of most flowering plants is composed of seven cells with different biological functions. Stressing the functional differentiation of cells of the embryo sac the idea of a female germ unit (FGU) was introduced in the 1980s, understood as the minimal set of cells required for correct and effective double fertilization. In typical representatives of gymnosperms, the FGU is composed of the egg apparatus (the egg cell and two synergids) and the central cell (Dumas and Mogensen 1993; Russell 1993). Synergids attract the pollen tube, and one of them becomes the site of delivery of sperm cells, which will participate in the process of double fertilization. The embryo develops from the fertilized egg cell and the endosperm nutritive tissue from the central cell. The remaining three cells of the female gametophyte are the antipodal cells, which have somatic roles and, in general, have already begun to degenerate before fertilization (Huang and Russell 1992; Grossniklaus and Schneitz 1998; Higashiyama 2002, 2003).

Morphological and structural investigations of the female gametophyte and the fertilization process have been performed in several species of plants, including maize (Diboll 1968; Heslop-Harrison et al. 1999), barley (Mogensen 1982), *Plumbago* (Russell 1985), *Populus* (Russell et al. 1990), and *Amaranthus* (Coimbra and Salema 1999), and also in vitro in *Zea* (Faure et al. 1994; Mól et al. 1994; Kranz et al. 1995) and wheat (Kumlehn et al. 1999). Based on histochemical and ultrastructural investigations, the *Angiospermae* egg cell is considered to be relatively inactive. In contrast, synergids are very active cells, which produce and secrete attractants during the maturation period of the flower that guides the pollen tube to the female gametophyte (Higashiyama et al. 2001) and likely contains factors that control the cessation of pollen tube growth, pollen tube discharge and gamete fusion (Weterings and Russell 2004).

This strong differentiation in the metabolic activity of (FGU) cells has been confirmed using FISH and immunocytochemical techniques (Pięciński et al. 2008; Niedojadło et al. 2011). The immunocytochemical localization of nascent transcripts in the cells of the *H. orientalis* mature embryo sac has indicated that the egg cell has a very low level of transcriptional activity and that only a slightly higher level of transcription occurs in the central cell. In contrast, the level of synthesis of new RNA in synergids is high, comparable to that in the nucellus cells surrounding the gametophyte (Niedojadło et al. 2011). The differentiated FGU transcriptional activity is in agreement with the level of poly(A) RNA and transcriptional factors present there (Pięciński et al. 2008). However, in an immature gametophyte, all cells of the embryo sac have a similarly high level of transcriptional activity (Niedojadło et al. 2011). Among newly synthesized transcripts, a large percentage probably consists of rRNA. Investigations in *Zea* have shown that, during the development of the embryo sac, the egg apparatus and the central cell contain severalfold more ribosomes than are present in the antipodal cells or the cells of the surrounding nucellus (Dow and Mascarenhas 1991a). A similar differentiation in the ribosome pool is maintained in the mature embryo sac when the egg cell is metabolically silenced (Dow and Mascarenhas 1991b). These data have suggested that ribosomes synthesized in the egg and central cell during the maturation of the embryo sac accumulate in their cytoplasms and may be used for translation after fertilization.

Ribosome biogenesis occurs in the nucleolus forming around the nucleolar organizer regions (NORs). The NOR contains rDNA encoding three of the four rRNAs of the large (LSU) and small (SSU) ribosomal subunits. The maturation of ribosomal subunits is a complex multistage process requiring the coordinated expression of pre-rRNA by RNA Pol I, ribosomal proteins by RNA Pol II and 5S rRNA by RNA Pol III. Pre-rRNA transcription, rRNA maturation and the assembly of rRNA and ribosomal proteins into preribosomal particles (RNPs) take place in the nucleolus. These complex processes are reflected in the structure of the nucleolus: the fibrillar centres (FC) are the site of occurrence of rDNA and factors involved in rRNA transcription, the fibrillar component (DFC) contains nascent transcripts and rRNA in the initial stages of maturation, and preribosomes are assembled in the granular component (GC) (reviewed in Carmo-Fonseca et al. 2000; Raška et al. 2006; Hernandez-Verdun 2006; Boisvert et al. 2007; Sirri et al. 2008). The primary 35-47S rRNA transcript contains two external transcribed spacers—5′ETS and 3′ETS, and two internal transcribed spacers, ITS1 and ITS2, in addition to the sequences of the ribosomal subunits. Pre-rRNA undergoes gradual cleavage by exo- and endonucleases, which leads to the successive removal of ETS, ITS1 and ITS2 and the formation of functional 18S rRNA, 28S rRNA (25S in *Saccharomyces cerevisiae*) and 5.8S rRNA (Raška et al. 2006; Strunk and Karbstein 2009). The cleavages are accompanied by additional cotranscriptional modifications of specific nucleotides in rRNA, the most common being 2′-*O*-ribose methylation and pseudouridylation (Brown and Shaw 1998). Short RNA molecules, called small nucleolar RNAs (snoRNAs), take part in this process. The best-characterized snoRNA required during the first stages of pre-rRNA maturation is the U3 snoRNA belonging to the C/D class of snoRNAs. In plants U3 snoRNA is transcribed by RNA Pol III, in contrast to animals in which RNA Pol II participates in this process (Kiss et al. 1991). U3 snoRNA contains a sequence complementary to the 5′-EST region and the 18S rRNA sequence and plays a crucial role in the first stages of pre-rRNA maturation and modification (among others Tollervey and Kiss 1997; Brown and Shaw 1998; Kiss 2002; Borovjagin and Gerbi 2005; Matera et al. 2007).

Functional rRNA transcripts form complexes with ribosomal proteins and are assembled into the preribosomal subunits. The 28S and 5.8S along with the 5S rRNA, which is synthesized in the nucleoplasm, will be part of the LSU, whereas the 18S rRNA will compose the SSU (Raška et al. 2004; Olson and Dundr 2005).

The multistage biogenesis of ribosomal subunits is a rigorously controlled process. The checkpoints are believed to be the successive stages of pre-rRNA cleavage and maturation (Strunk and Karbstein 2009; Deisenroth and Zhang 2010). Experimental analysis, mainly in yeast, has shown that impaired rRNA synthesis, perturbations to rRNA modification and processing, or RNP imbalance can all disrupt ribosome biogenesis. Recent investigations have demonstrated that molecules that control the downstream events of rRNP maturation are present in the processosome responsible for assembling the ribosomal subunits. These molecules probably physically block the attachment of ribosomal proteins and other maturation factors. The incorporation of factors necessary for the subsequent steps of rRNP assembly is only possible after their removal (Connolly et al. 2008; Strunk and Karbstein 2009).

Ribosome biogenesis requires coordination with other cellular processes, such as stress response, growth state and cell cycle progression. The disintegration of the nucleolus during cell division is a well-known phenomenon. Investigations in mice have shown that the nucleolar metabolism also affects the interphase process. The expression of the mutated form of the Bop1 protein, which is involved in rRNA processing, blocks the formation of 28S rRNA and 5.8S rRNA and inhibits the cell cycle (Pestov et al. 2001). Further investigations have demonstrated that, in human cells, perturbations of rRNA processing activate the p53 protein (Deisenroth and Zhang 2010), which is responsible for arresting the cell cycle, among other things (Levine et al. 2006). On this basis, it is believed that factors involved in nucleolar RNA maturation may transfer the signal to p53 through mediators and, in this way, affect the progression of the cell cycle.

In most diploid flowering plants, one large nucleolus is present in the mature egg cell. Male gametes, both in the tricellular pollen grain in *Zea mays* (McConchie et al. 1987; Mogensen et al. 1990) as well as in *H. orientalis* (Zienkiewicz et al. 2011), in which sperm cells are only formed in the pollen tube, do not have this structure. These data indicate that the paternal rRNA genes are inhibited before fertilization, and the paternal partner probably does not contribute rRNPs to the zygote. Cytological investigations in *Zea* have shown that the nucleolus of the egg cell has already become active already 1 h after IVF, whereas the second nucleolus, showing activation of the paternal NOR, is only visible 13–18 h after IVF (Faure et al. 1994; Mól et al. 1994; Kranz et al. 1995). During this period in the maize zygote the levels of the transcripts of the ribosomal proteins S21A and L39 are also increased (Dresselhaus et al. 1999a), which may suggest a resumption of ribosome maturation.

The appearance of the second nucleolus within the zygote and a third one in the primary endosperm cell may be a good tool for checking whether zygotic gene activation (ZGA) has already taken place during the first post-fertilization interphase. Our investigations in *H. orientalis* have shown that, shortly after fertilization in vivo, a strong activation of transcription takes places in the zygote and endosperm, which is accompanied by an increase in the level of RNA Pol II (Niedojadło et al. 2011) and the synthesis and processing of mRNA (Pięciński et al. 2008). However, little is known about rRNA metabolism both in the target cells for male gametes and in the synergids and antipodal cells of the embryo sac.

The aim of our present investigations was to analyze in vivo the nucleolar activity of the cells of the mature embryo sac during the rarely studied progamic phase and after fertilization. Using fluorescence in situ hybridization (FISH), the distributions of the newly formed pre-rRNA (ITS1), 26S rRNA, 5S rRNA and U3 snoRNA were determined in the cells of the female gametophyte of *Hyacinthus orientalis* L. These methods allowed us to demonstrate the differentiated rRNA metabolism of the *H. orientalis* embryo sac cells before and after fertilization.

## Materials and methods

### Material for investigations

*Hyacinthus orientalis* L. (commercial cultivar has grown at room temperature in the Institute of General and Molecular Biology, Nicolaus Copernicus University, Toruń, Poland) ovules before and after fertilization were used. Pistils were hand cross-pollinated and after 8, 24, and 96 h ovules were mechanically isolated from the flowers. The growth of the pollen tubes was checked by isolated pistils which were cut up, placed in 0.01% aniline blue and were examined using the fluorescence microscopy.

### Sample preparation

Directly after isolation ovules were fixed in 4% paraformaldehyde (Polyscience) and 0.25% glutaraldehyde (Sigma) in phosphate-buffered saline (PBS) buffer pH 7.2 overnight at 4°C. The material was dehydrated in increasing ethanol concentrations containing 10 mM dithiothreitol (DDT) (Fermentas), supersaturated and then embedded in BMM resin (butyl methacrylate, methyl methacrylate, 0.5% benzoil ethyl ether (Fluka) with 10 mM DDT) at −20°C under UV light for polymerization. The embedded material was cut on Leica UCT ultramicrotome into semithin sections (1.5 μm), which were placed on microscope slides coated with Biobond (British Biocell).

### Fluorescence in situ hybridization

Using the oligonucleotide probes ITS1 pre-rRNA (5′-Cy3-ACG GGT TCG CGA TCG TCC GTT CGG G-3′), 26S (5′-Cy3-AGC TAC TAG ATG GTT CGA TTA GTC TTT C-3′), 5S (5′-Cy3-GGT GCA TTA ACG CTG GTA TGA TC-3′), U3 snoRNA (5′-Cy3-GTA CGA GCC TAT AGA ACA GAT CCT TTC AA GTA AGG TCG T-3′) were detected (GenoMed, Warsaw, Poland). For hybridization, the probes were resuspended in hybridization buffer [30% formamide, 4× SSC, 5× Denhart’s buffer, 1 mM EDTA, 50 mM phosphate buffer, H_2_O (Sigma)] from 100 to 200 pmol/ml. Hybridization was performed for 12 h at 37°C. DNA was stained with DAPI (Fluka). ITS1 pre-rRNA and 26S rRNA were detected on serial sections of the embryo sac. The positive and negative control reactions were performed using somatic cell samples. For the negative control of reactions the samples were incubated with hybridization buffer, omission of the probe (Supplemental data).

### Quantitative evaluations

Image analysis was performed on serial semithin sections after FISH (ITS1 pre-rRNA, 26S rRNA, 5S rRNA, U3 snoRNA), with each reaction step performed using consistent values of temperature, incubation times and probe concentrations. The quantitative analysis of fluorescence intensity was carried out for 5–7 each cell types (five sections per cell) from each developmental stage. The observations of the FISH experiments were carried out with Olympus BX50 fluorescence microscope. The UPlanFI 100× (N.A. 1.3) oil immersion lens and narrow band filters (U-MNU, U-MNG) were used. The results were registered with a Olympus XC50 digital colour camera and Cell^B^ software (Olympus Soft Imaging Solutions GmbH, Münster, Germany). All measurements were conducted at the same magnification, field area (controlled with a shutter) and positioning of the fibre optics cable. The camera settings were kept constant for exposition time, gain and offset. Cell^B^ software was used to determine the average μm^3^ signal intensity of each studied cell compartment and is expressed in a.u. For all probes and developmental stages, the obtained data were corrected for background autofluorescence as determined from negative control signal intensities. To test differences among multiple samples (i.e., levels of signal in different stages), a Kruskal–Wallis ANOVA test was used. Statistical data and graphs were created using Microsoft Excel 2007 software. The error bars indicate one standard error (SE) of the mean.

## Results

### rRNA localization

#### Mature embryo sac

**Fig. 1:**
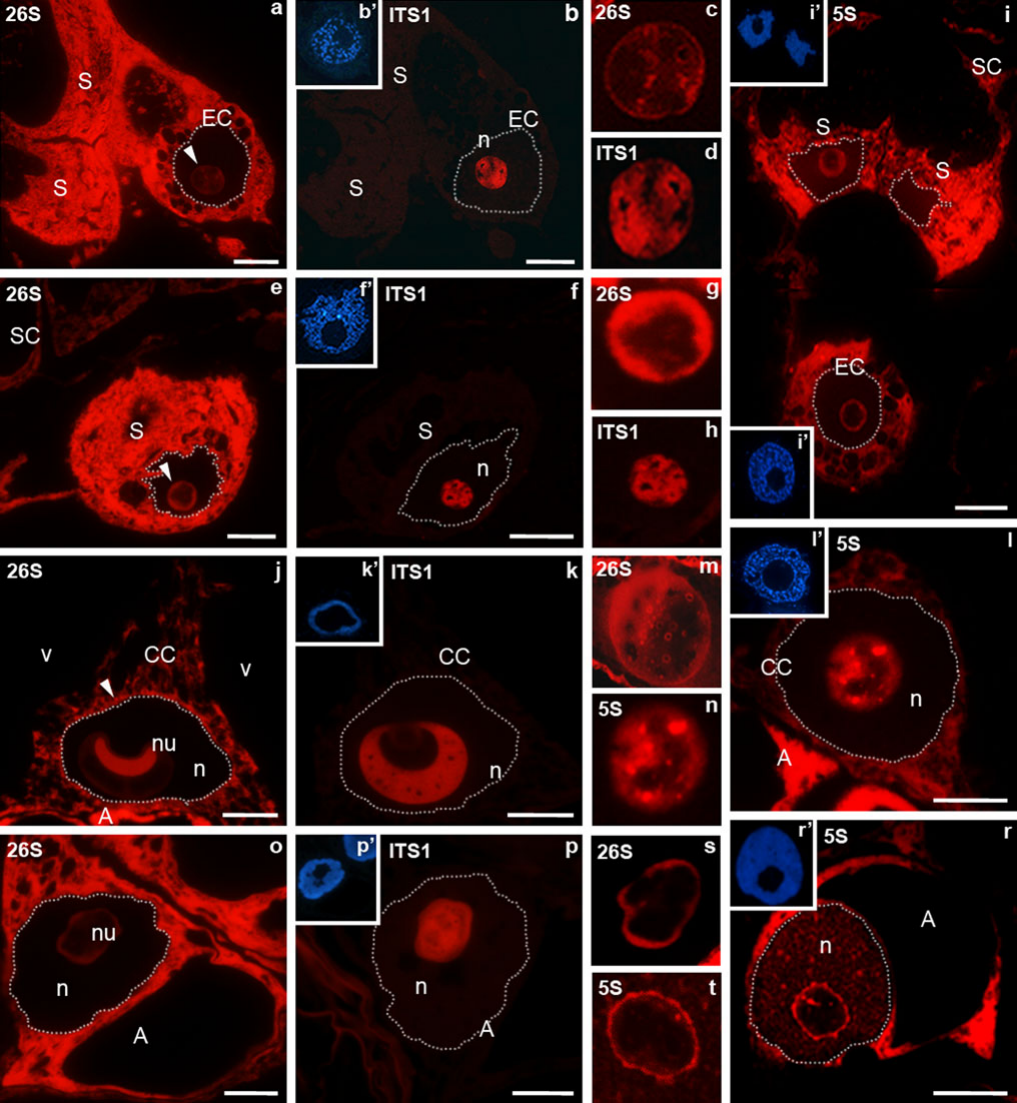
Localization of ITS1 pre-rRNA, 26S rRNA, 5S rRNA in *H. orientalis* mature embryo sac. Egg apparatus (**a**–**i**). The high fluorescence 26S rRNA is present in the egg cell and the synergids cytoplasm. In the egg cell nucleolus (*arrow*) the fluorescence intensity is very low (**a**). ITS1 pre-rRNA strong labelling is present throughout the nucleolus (**b**). Increased signal of 26S rRNA is seen around large areas (**c**) in which ITS1 pre-rRNA is present (**d**). The labelling of 26S rRNA in the synergid cytoplasm is higher than in that the somatic cell (**e**). The level of 26S rRNA in the synergid nucleolus (*arrow*) is higher than in the egg cell nucleolus (compare **a**). Increased signal is localized in periphery part of the nucleolus (**g**) around the central part containing ITS1 pre-rRNA (**f**, **h**). The high 5S rRNA labelling (higher than in the somatic cell) is present in the synergid and the egg cell cytoplasm. In these cells the signal of fluorescence is also localized in the peripheral part of the nucleolus (**i**). Central cell (**j**–**l**). 26S rRNA is present in the cytoplasm but the higher fluorescence is visible in the boundary between the nucleus and the cytoplasm (*arrow*) (**j**). In the nucleolus the high signal is seen around area which ITS1 pre-rRNA is localized (**k**). 5S rRNA is localized in the cytoplasm and in dispersed or cluster form in the nucleolus (**l**). 26S rRNA signal correspond to the areas of 5S rRNA in the nucleolus (**m**, **n**). A very high signal of 26S RNA and 5S rRNA is present in the antipodal cell (**o**, **r**). In those nucleolus the signal of fluorescence is localized in periphery part whereas in central part ITS1 pre-rRNA is present (**p**, **s**, **t**). *b*′, *f*′, *i*′, *k*′, *l*′, *p*′, *r*′ DAPI staining. ITS1 pre-rRNA and 26S rRNA were detected on serial sections of the embryo sac, so *b*′, *f*′, *k*′, *p*′ DAPI images are for both reactions. *A* antipodal call, *CC* central cell, *EC* egg cell, *n* nucleus, *S* synergid, *SC* somatic cell, *v* vacuole. *Bar* 10 μm

In the cells of the egg apparatus, high signals for 26S rRNA and 5S rRNA were observed in the cytoplasm, outside the area of the vacuoles and amyloplasts (Fig. 1a, i). The labelling of the egg apparatus cytoplasm was distinctly higher than that found in the somatic cells surrounding the embryo sac (Fig. 1e). However, the cells of the egg apparatus differed in the level of the signal from the nucleolus. In the nucleolus of the egg cell, 26S rRNA labelling was very low (Fig. 1a); however, a high signal of ITS1 pre-rRNA was observed there (Fig. 1b). The comparison of the localization of 26S rRNA and ITS1 pre-rRNA in serial sections showed that an increased signal of 26S rRNA was localized around large areas in which ITS1 pre-rRNA was present in the nucleolus of the egg cell (see Fig. 1c, d). 5S rRNA was observed mostly at the rim of the nucleolus (Fig. 1i). In the synergid nucleolus, the level of 26S rRNA labelling was higher than in the egg cell (Fig. 1e). Within this nucleolus, a strong fluorescence was also observed, indicating the presence of ITS1 pre-rRNA (Fig. 1f). Increased 26S rRNA labelling was localized in the form of a broad belt around the central part containing ITS1 pre-rRNA (Fig. 1g, h). A high signal of ITS1 pre-rRNA was found in the form of “hoops” that surrounded small oval areas lacking fluorescence. The peripheral part of the synergid nucleolus was also the area in which 5S rRNA was localized (Fig. 1i). In the central cell, similar to the signal observed in the cells of the egg apparatus, the 26S rRNA (Fig. 1j) and 5S rRNA (Fig. 1l) signals were localized in the cytoplasm. A particular accumulation of 26S rRNA was observed at the boundary between the nucleus and the cytoplasm (Fig. 1j). In the nucleolus of the central cell, the fluorescence intensity indicating the presence of 26S rRNA was higher than in the egg cell (see Fig. 1c, m). In the area of the large nucleolus of this cell, segregation of an area with elevated 26S rRNA labelling from an area containing ITS1 pre-rRNA was sometimes observed. The area with a very strong fluorescence indicating 26S rRNA (Fig. 1j) was surrounded by an almost homogeneous signal of ITS1 pre-rRNA (Fig. 1k). In general, the 26S rRNA signal was, however, observed almost in the entire area of the nucleolus of the central cell, with the fluorescence localized on its rims and in the form of larger and smaller clusters in the central region (Fig. 1m). The sites of the increased 26S rRNA signal (Fig. 1m) corresponded to the areas of 5S rRNA occurrence (Fig. 1n). A very high signal of 26S rRNA (Fig. 1o) and 5S rRNA (Fig. 1r) was also observed in the cytoplasm of the antipodal cells. A much lower fluorescence derived from these molecules was observed in the nucleolus of these cells. The increased 26S rRNA (Fig. 1s) and 5S rRNA (Fig. 1t) signals were mainly localized at the boundaries of the nucleolus, whereas the central part was labelled with the ITS1 pre-rRNA probe (Fig. 1p). Small clusters of fluorescence indicating the presence of 5S rRNA were also observed in o the nucleoplasm of antipodal cells (Fig. 1r).

#### Progamic phase

**Fig. 2:**
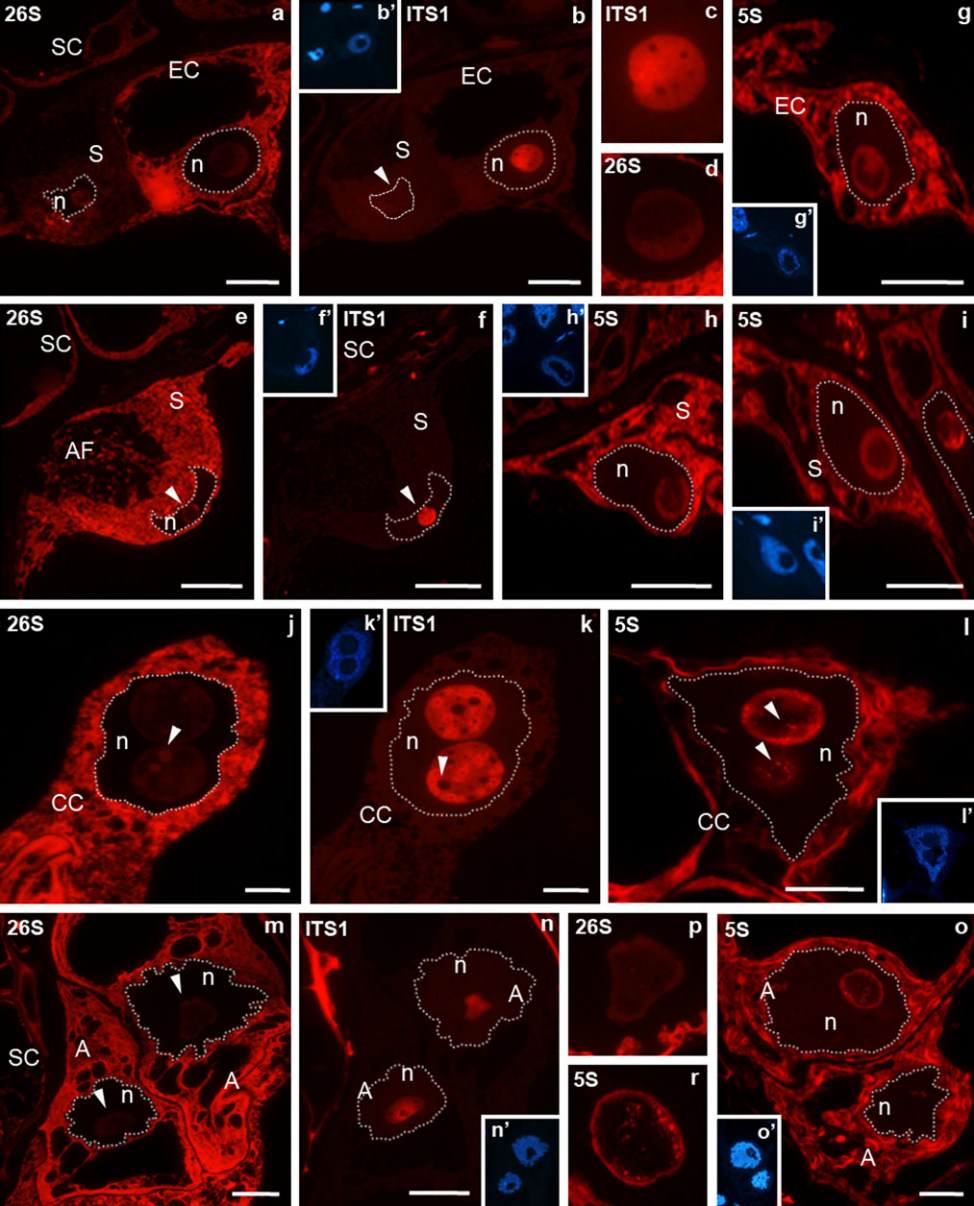
Localization of ITS1 pre-rRNA, 26S rRNA, 5S rRNA in *H. orientalis* embryo sac in the progamic phase. Egg apparatus (**a**–**i**). In the egg cell 26S rRNA and 5S rRNA signals are high in the cytoplasm and very low in the nucleolus (**a**, **g**). ITS1 pre-rRNA (**b**, **c**) is surrounded by low 26S rRNA labelling in the nucleolus (**d**). In the synergid 26S rRNA signal is lower than in the egg cell cytoplasm, the low level of fluorescence is seen in the nucleolus (**a**). ITS1 pre-rRNA (*arrow*) is not visible in the synergid nucleolus (**b**). In the second synergid (**e**) 26S rRNA labelling is higher (compare **a**) in the cytoplasm and very low level of fluorescence is seen in the nucleolus (*arrow*). The homogenous fluorescence indicating the presence of ITS1 pre-rRNA is present in the nucleolus (**f**). Synergids in the same egg apparatus. 5S rRNA labelling in one synergid is higher than that in the somatic cells (**h**) in the second synergid is similar to that observed in the somatic cells (**i**). **j**–**l** Central cell with two nucleoli. The high 26S labelling (**j**) is present in the cytoplasm and in the periphery part and irregular clusters in the nucleolus (*arrow*). The homogenous fluorescence in the central part and a small circular areas in periphery part devoid (*arrow*) of ITS1 pre-rRNA labelling are seen in the nucleolus (**k**). 5S rRNA is present in the cytoplasm in the periphery part and in clusters (*arrow*) in the nucleolus of the central cell (**l**). In the antipodal cells the high 26S rRNA and 5S rRNA signals of fluorescence are in the cytoplasm (**m**, **o**). In the nucleolus these signals are present in the peripheral part (**p**, **r**). ITS1 pre-rRNA is localized in the central part of the nucleolus (**n**). *b*′, *f*′, *g*′, *h*′, *i*′, *k*′, *l*′, *n*′, *o*′ DAPI staining. ITS1 pre-rRNA and 26S rRNA were detected on serial sections of the embryo sac, so *b*′, *f*′, *k*′, *n*′ DAPI images are for both reactions. *A* antipodal cell, *CC* central cell, *EC* egg cell, *n* nucleus, *S* synergid, *SC* somatic cell. *Bar* 10 μm

During the progamic phase in the egg cell, the 26S rRNA (Fig. 2a) and 5S rRNA (Fig. 2g) signal were still high in the cytoplasm and low in the nucleolus. These molecules were localized predominantly in the peripheral area of the nucleolus, similar to the localization observed before pollination (Fig. 2d, g), whereas ITS1 pre-rRNA (Fig. 2b, c) was present in the central area. Changes in the distribution and in the level of rRNA were, however, noted in synergids. Some cells were observed in which the cytoplasmic 26S rRNA (Fig. 2a) and 5S rRNA (Fig. 2i) signal were very low, while the cytoplasm of others was very strongly labelled (Fig. 2e, h). The nucleolus of a synergid with a very low cytoplasmic 26S rRNA signal was almost completely devoid of ITS1 pre-RNA labelling (Fig. 2b). Fluorescence of ITS1 pre-rRNA was only observed in the nucleolus of a synergid, in which a very high cytoplasmic 26S rRNA signal was observed (Fig. 2b). In the central cell, stronger labelling of 26S rRNA (Fig. 2j) and 5S rRNA (Fig. 2l), than that observed before the pollination, was observed in the cytoplasm surrounding the cell nucleus. In large nucleoli, the 26S rRNA signal (Fig. 2j) was lower than that observed before pollination, with fluorescence localized at the rim and in the form of irregular clusters in the central. A similar distribution was found for the 5S rRNA signal (Fig. 2l). Both nucleoli were strongly labelled by ITS1 pre-rRNA (Fig. 2k). The pre-rRNA signal was homogenous, with a few small areas devoid of fluorescence (Fig. 2k). The analysis of serial sections indicated that these areas corresponded to clusters labelled with 26S rRNA. High 26S rRNA (Fig. 2m) and 5S rRNA (Fig. 2o) signals, higher than those in somatic cells, were also observed in the cytoplasm of antipodal cells. In the nucleolus of antipodal cells, the 26S rRNA signal decreased and was lower than that observed before pollination (for comparison Fig. 1o). The distribution of the analysed rRNA signal in the nucleolus of the antipodal cells did not change significantly. Fluorescence indicating ITS1 pre-rRNA filled the central part of the nucleolus (Fig. 2n), whereas increased labelling 26S rRNA (Fig. 2p) and 5S rRNA signals (Fig. 2r) were mainly localized at its peripheral part. Only single clusters of the 26S rRNA and 5S rRNA signals were present in the central part of the nucleolus. In contrast to the period before pollination, no 5S rRNA signal was observed in the nucleoplasm of the antipodal cells (Fig. 2o).

#### After fertilization

**Fig. 3:**
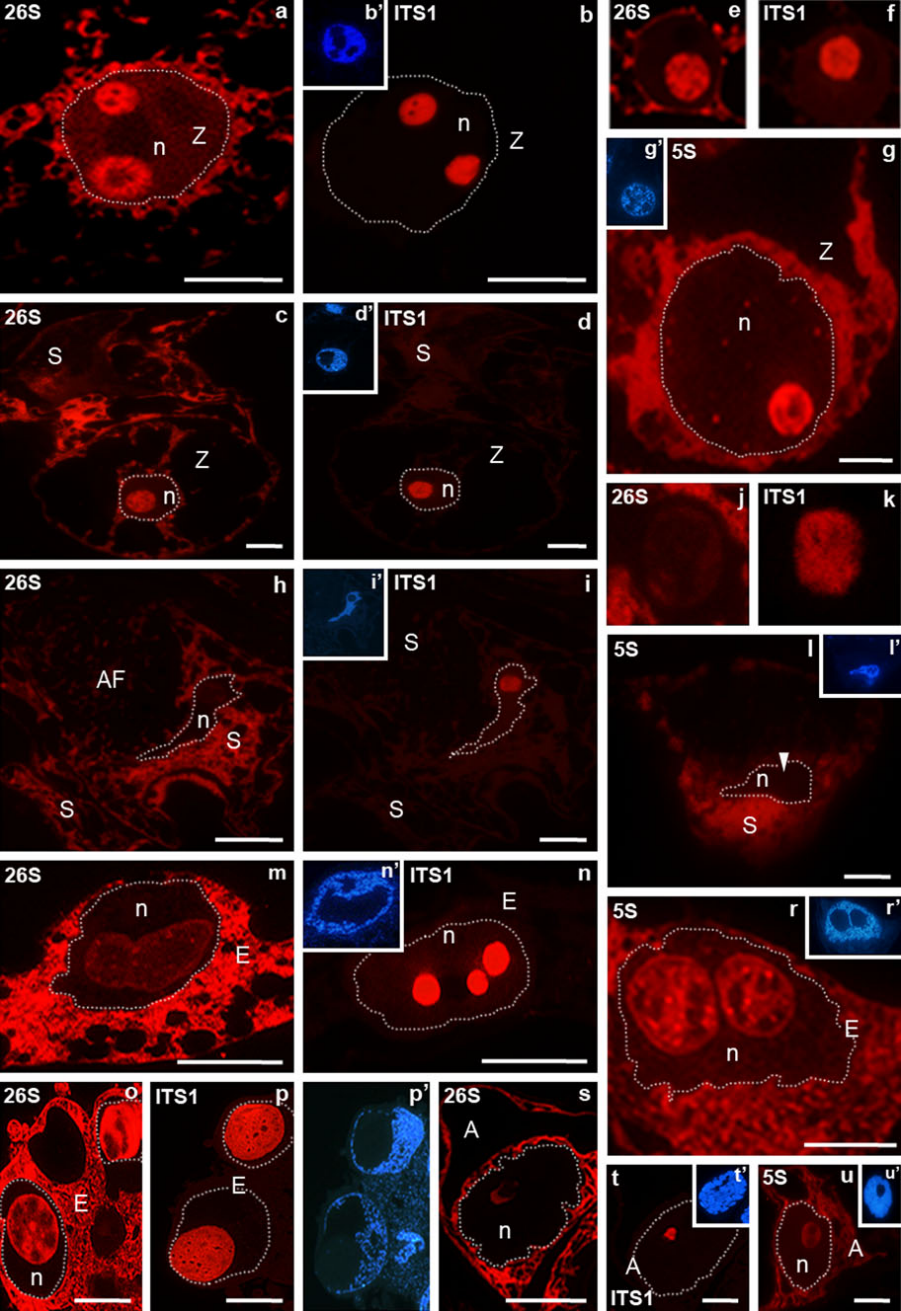
Localization of ITS1 pre-rRNA, 26S rRNA, 5S rRNA in *H. orientalis* fertilized embryo sac. In the zygote with two nucleoli (**a**, **b**) in one of them (smaller) 26S rRNA and ITS1 pre-rRNA signals are similar. In the second nucleolus, strong 26S rRNA labelling in the periphery part (**a**) and ITS1 pre-rRNA in the central part are present (**b**). In the zygote with one nucleolus the high level of 26S rRNA is in the cytoplasm and in the nucleolus (**c**). 26S rRNA (**e**) and ITS1 pre-rRNA (**f**) are present throughout the nucleolus. ITS1 pre-rRNA signal of fluorescence is high (**d**). 5S rRNA is present in the cytoplasm and in the nucleolus. The clusters of signal of fluorescence are present in the nucleoplasm (**g**). In the synergid the higher labelling of 26S rRNA (**h**) and 5S rRNA (**l)** is localized in the perinuclear cytoplasm. In the nucleolus these signals are very low or not observed (**h**, **j**, **l**). ITS1 pre-rRNA is still present (**i**, **k**). **m**–**p** Endosperm. 26S rRNA fluorescence is present in the cytoplasm and in the nucleoli (**m**, **o**). ITS1 pre-rRNA is observed in the nucleoli (**n**) and higher level is present in the form of “hoops” around small areas devoid the fluorescence (**p**). 5S rRNA is present in the cytoplasm and in the periphery part and in clusters in central part of the nucleoli (**r**). In the degenerating antipodal cells decreased levels of all signals are observed (**s**–**u**). *b*′, *d*′, *g*′, *i*′, *l*′, *n*′, *p*′, *r*′, *t*′, *u*′ DAPI staining. ITS1 pre-rRNA and 26S rRNA were detected on serial sections of the embryo sac, so *b*′, *d*′, *i*′, *n*′, *p*′ DAPI images are for both reactions. *A* antipodal cell, *AF* filiform apparatus, *E* endosperm cell, *n* nucleus, *S* synergid, *SC* somatic cell, *Z* zygote. *Bar* 10 μm

Fertilization induced changes in the levels of the analysed molecules in the *H. orientalis* embryo sac cells (Fig. 5). Within the zygote, strong labelling of rRNA was localized both in the cytoplasm as well as in the area of one or two nucleoli (Fig. 3a–g). In the zygote with two nucleoli, the localization of the 26S rRNA (Fig. 3a) and ITS1 pre-rRNA (Fig. 3b) signals was very similar in one of the nucleoli: the whole nucleolus was labelled with the exception of small areas. In the second nucleolus, strong labelling of 26S rRNA occurred mainly in its peripheral part, whereas the fluorescence was weaker in the central part in which the ITS1 pre-rRNA is located (Fig. 3a, b). In the case of a zygote with one nucleolus the 26S rRNA (Fig. 3c) and 5S rRNA (Fig. 3g) signals were present throughout the nucleolus and in the central part that had a strong ITS1 pre-rRNA (Fig. 3d). The 5S rRNA signal was also localized in the area of the zygote nucleoplasm and presented as individual small clusters (Fig. 3g). Levels of all studied molecules were higher compared with those observed before fertilization in the egg cell. Particularly, significant increase (approximately 100-fold) of the level of mature 26S rRNA was noted in the nucleoplasm of the zygote with twice the signal of fluorescence increases in the nucleolus (Fig. 5). After fertilization in some synergids, labelling of 26S rRNA (Fig. 3h) and 5S rRNA (Fig. 3l) was also observed in the perinuclear cytoplasm. In the nucleolus of such a synergid, labelling of 26S rRNA (Fig. 3j) and 5S rRNA (Fig. 3l) was almost completely absent, even though the ITS1 pre-rRNA signal could still be observed in it (Fig. 3i, k). In the primary endosperm cell, a high 26S (Fig. 3m) and 5S rRNA (Fig. 3r) signals were present in the perinuclear cytoplasm. In the large nucleolus with an irregular shape, the relatively weak labelling of 26S rRNA was observed throughout its whole volume, with increased fluorescence observed mainly on its rim and small clusters in its central part (Fig. 3m). The analysis of the ITS1 pre-rRNA signal in a series of sections of this cell indicated that pre-rRNA occurs in the form of three large oval structures in a single nucleolus organized in this fashion (Fig. 3n). The nucleolar signal of 5S rRNA in the primary endosperm cell was heterogeneous, dispersed at its rims and present in the form of clusters in the central part (Fig. 3r). In turn, the labelling of 26S rRNA was high in large single nucleoli of endosperm with several nuclei. A strong signal was localized mainly at the rims of the nucleolus and in the central part around oval areas with diminished fluorescence (Fig. 3o). In these nucleoli, the labelling of ITS1 pre-rRNA was also high, with fluorescence present in nearly the entire area (Fig. 3p). An increased level of signal was observed in the form of “hoops” around small structures lacking fluorescence. A clear decrease in the labelling of all analysed molecules was observed in the degenerating antipodal cells. Decreased 26S rRNA (Fig. 3s) and 5S rRNA (Fig. 3u) signals were observed in the cytoplasm and at the periphery of the nucleolus. In its central part, a weak ITS1 pre-rRNA signal was still present (Fig. 3t).

### U3 snoRNA

#### Mature embryo sac

**Fig. 4:**
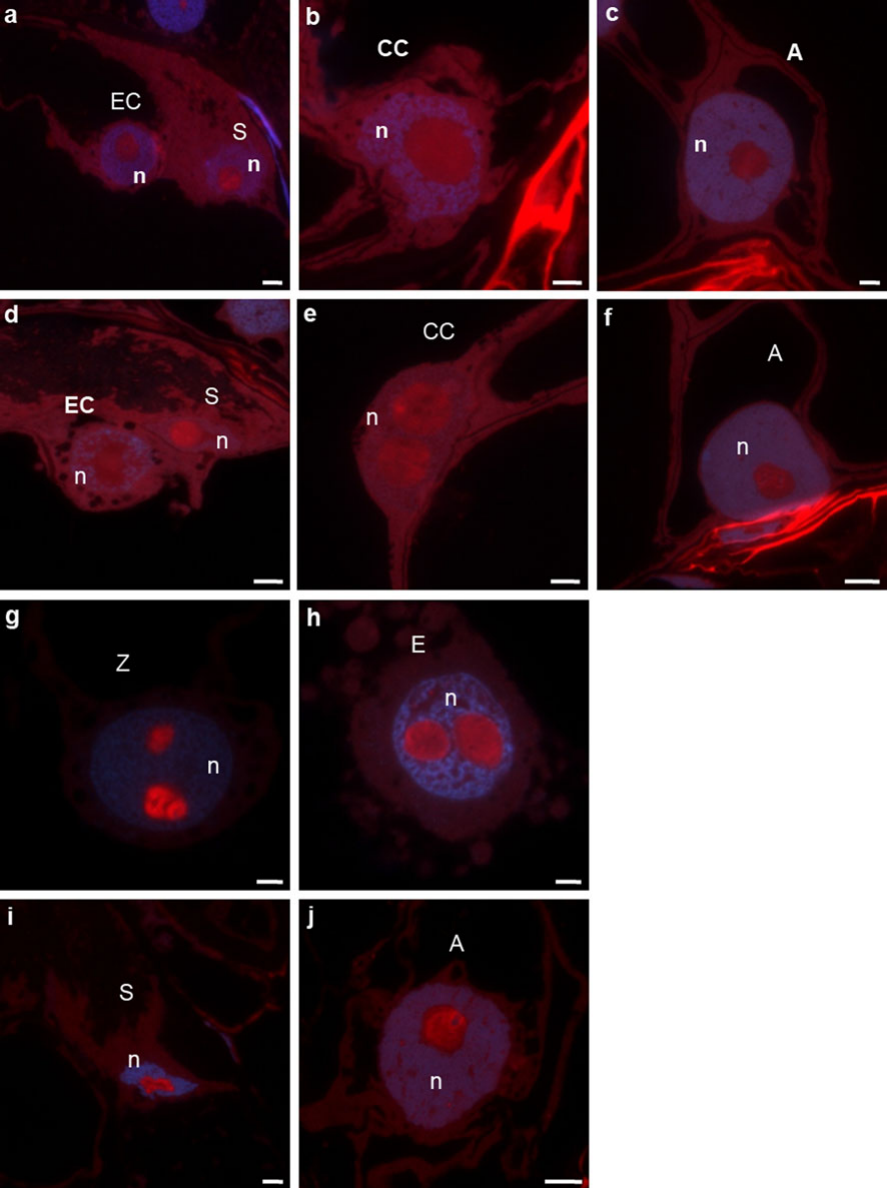
Localization of U3 snoRNA in the *H. orientalis* embryo sac. **a**–**c** Mature embryo sac. In the synergid the higher labelling than in the egg cell is present (**a**). In the central cell the signal is observed throughout the nucleolus (**b**) but in the antipodal cell in the central part of the nucleolus (**c**). **d**–**f** Progamic phase. In the egg cell low and heterogenous signal is localized. The labelling of synergid is homogenous and higher than that observed before pollination (**d**). In the central cell (**e**) and in the antipodal cell (**f**) the strong and heterogenous fluorescence is present. **g**–**j** Fertilized embryo sac. The high labelling is observed in both zygote nucleoli (**g**). In the endosperm strong fluorescence in the nucleoli and in clusters in the nucleoplasm is present (**h**). The weak signal is still observed in the degenerating synergid (**i**) and in the antipodal cell (**j**). *blue* DAPI staining. *A* antipodal cell, *E* endosperm cell, *n* nucleus, *S* synergid, *SC* somatic cell, *Z* zygote. *Bar* 10 μm

A differentiated distribution of U3 snoRNA was found in the cells of the egg apparatus. The labelling of the nucleolus of the egg cell was very low, clearly lower than in the synergid nucleolus (Fig. 4a). In the synergid, the signal was localized almost throughout the whole volume of the nucleolus, even though areas of stronger and weaker fluorescence were observed there. In the central cell, a relatively high U3 snoRNA signal was observed in the form of numerous clusters and, similarly to ITS1 pre-rRNA, sometimes localized in only one part of the nucleolus (Fig. 4b, compare with Fig. 1k). Within the nucleoli of the antipodal cells, the signal was present in the central part. This signal was mainly localized within the “hoop” surrounding areas that lacked labelling (Fig. 4c).

#### Progamic phase

After the pollination of the pistil, fluorescence indicating the presence of U3 snoRNA in the nucleolus of the egg cell was almost invisible. In contrast, the labelling of the synergid nucleolus remained high, with signal observed throughout almost its entire area (Fig. 4d). In contrast to the egg cell, the signal was relatively high in both nucleoli of the central cell. In these nucleoli, the signal was mainly observed in the form of irregular “hoops” in the central region of the nucleolus (Fig. 4e). In the antipodal cells, considerable labelling of nucleoli was detected. A heterogeneous signal was observed throughout almost the entire volume (Fig. 4f).

#### After fertilization

**Fig. 5:**
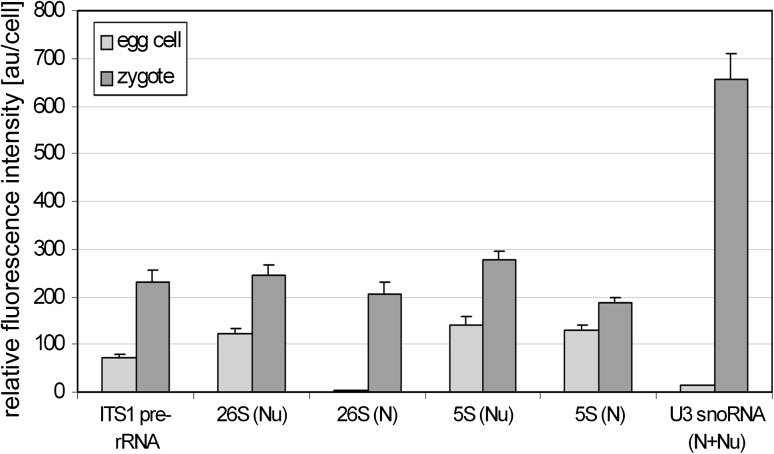
*Graph* comparing the levels of ITS1 pre-rRNA, 26S rRNA, 5S rRNA and U3 snoRNA in *H. orientalis* egg cell and zygote. The intensities of fluorescence indicating all molecules were significantly higher after fertilization. *N* nucleoplasm, *Nu* nucleolus. For statistical information, see “Materials and methods”

In both nucleoli of the zygote, U3 snoRNA labelling was very high (Fig. 4g). A strong fluorescence was observed almost in the whole area of the nucleoli, and an especially high signal was observed in the form of thick “hoops” filling the central part of the nucleolus. The level of U3 snoRNA increased over 40-fold in comparison with the level which was observed in the egg cell before fertilization (Fig. 5). Similar to the zygote, a high U3 snoRNA signal was observed in endosperm nucleoli. A strong, nearly homogeneous fluorescence was observed throughout their entire volume. Signal foci also occurred in the nucleoplasm (Fig. 4h). A relatively weak signal was still visible in the nucleoli of degenerating synergids (Fig. 4i) and antipodal cells (Fig. 4j).

#### Control reactions

In the somatic cells of negative control reactions for hybridizations, without labelled probes complementary to ITS1 pre-rRNA, 26S rRNA, 5S rRNA and U3 snoRNA, the lack of background was observed (Supplemental data, Fig. S1a). In positive controls for FISH, using the probes, the strong signals of the fluorescence were present. 26S rRNA (Suppl data, Fig. S1b) and 5S rRNA (Suppl data, Fig. S1d) labelling was observed in the cytoplasm and the nucleoli of the somatic cells. Signals of ITS1 pre-rRNA (Suppl data, Fig. S1c) and U3 snoRNA (Suppl data, Fig. S1e) were localized in the nucleoli of these cells.

## Discussion

Our results demonstrated that all of the FGU cells in *H. orientalis* contain a large pool of cytoplasmic rRNA. The mature egg cell of this species is known to be transcriptionally silenced, and only slightly higher transcription is found in the central cell (Niedojadło et al. 2011). In both cells, there is also a low level of poly(A) RNA (Pięciński et al. 2008), indicating that protein synthesis is also strongly limited. It is, therefore, reasonable to assume that most of the rRNPs present in the cytoplasm of the egg and central cell are not involved in translation and constitute a pool of inactive ribosomal subunits. A large rRNP pool is also present in the cytoplasm of the egg cell and the central cell in the progamic phase, when these cells are transcriptionally silenced (Niedojadło et al. 2011). These observations suggest that ribosomes that can be used for translation following fertilization are stored in the cytoplasm of these cells. rRNA synthesis, processing and assembly probably take place during the differentiation of the female gametophyte, when all of its cells are highly transcriptionally active (Niedojadło et al. 2011).

A high level of ribosomes in the cells of the embryo sac, including the egg cell, was observed earlier in *Zea* (Dow and Mascarenhas 1991a, b). Investigations using the in situ hybridization method with a probe for 25S rRNA indicated that the number of ribosomes increases during the development of the embryo sac, reaching the highest level during its maturity.

In *H. orientalis* a strong activation of RNA synthesis takes place shortly after fertilization in the zygote and primary endosperm cell, including mRNA synthesis (Niedojadło et al. 2011) and increased levels of poly(A) RNA and splicing factors (Pięciński et al. 2008). This reveals a strong resumption of the transcription of genes encoding proteins. Genetic analyses in several species, including *Zea* (Dresselhaus et al. 1999b; Scholten et al. 2002; Grimanelli et al. 2005), wheat (Sprunck et al. 2005) and *Arabidopsis* (Vielle-Calzada et al. 2000; Pagnussat et al. 2005; Pillot et al. 2010) indicate that transcripts are present in the egg cell that undergo translation only after fertilization. The expression of these early mRNAs, both synthesized before and after fertilization, probably takes place using the maternal translational apparatus synthesized during the maturation of the target cells for sperm cells.

In contrast, rRNA metabolism in synergids is in agreement with their differentiated activity in various stages of the embryo sac development. During anthesis synergids are highly transcriptionally active (Niedojadło et al. 2011), producing and secreting attractants for the pollen tubes (Berger et al. 2008). The presence of a large poly(A) RNA pool (Pięciński et al. 2008) and a well-developed ER (Huang and Russell 1992) indicate that intense translation is taking place. This result is in agreement with the large ribosome pool in the cytoplasm of the central cell and the presence of a presumably still active nucleolus, in which a large amount of U3 snoRNA was present in addition to pre-rRNA and 26S RNA. During the progamic phase in *H. orientalis* synergids, a progressive inhibition of transcription took place. This process was particularly visible in one of the cells (Niedojadło et al. 2011), which would probably become the target cell for the pollen tube (Russell 1996; Higashiyama 2002). The inhibition of activity was accompanied by a decrease in the ribosome pool until a nearly complete disappearance of rRNA from the cytoplasm occurred in one of the synergids. After fertilization, the degradation of cytoplasmic rRNA also took place in the second synergid, although immature rRNA transcripts were still present in many nucleoli.

The localizations of the rRNA synthesized in the NOR (26S rRNA, pre-rRNA) and in the nucleoplasm (5S rRNA) and of the nucleolar factor participating in pre-rRNA maturation (U3 snoRNA) have allowed a better understanding of the nucleolar activity of *H. orientalis* embryo sac cells. The localization of 26S rRNA in the nucleoli of cells from the unfertilized embryo sac was intriguing. 26S rRNA is known to occur in pre-rRNA as well as in the large ribosomal subunit. In the unfertilized embryo sac, however, the 26S rRNA mainly localized to the regions of the nucleolus, in which 5S rRNA, which is added to the large ribosomal subunit in the final stages of its maturation, was also present. This result indicates that the probe used mainly hybridized to the 26S rRNA sequence already after its excision from the primary transcript.

The nucleolus of the egg cell is the site of accumulation of large amounts of pre-rRNA. Transcripts hybridizing to the 26S rRNA probe were localized mainly at the rims of large areas containing pre-rRNA, particularly in the peripheral parts of the nucleolus, in which 5S rRNA was also present. The process of pre-rRNA maturation in the nucleolus of the egg cell appears to be slow as only a small amount of U3 snoRNA was localized there. Investigations using BrU have indicated that the nucleolus of the mature egg cell contains almost no nascent transcripts (Niedojadło et al. 2011), suggesting a strong silencing of rDNA genes. During the progamic phase, the pool of the detected 26S rRNA and U3 snoRNA in the nucleolus of the egg cell probably decreased even further as the signal from these molecules was almost invisible. However, the level of pre-rRNA remained very high. These observations allow us to propose that the nucleolus of the egg cell is the storage site for immature rRNA transcripts. If this is the case, then what are the factors that might participate in the inhibition of pre-rRNA maturation in the *H. orientalis* egg cell? Studies in other species, mainly yeast, have shown that rRNA processing and assembly are strongly controlled. The checkpoints are probably the successive stages of pre-rRNA cleavage and maturation (Strunk and Karbstein 2009; Deisenroth and Zhang 2010). Strunk and Karbstein (2009) believe that downstream events can be prevented by the binding of additional factors that physically block the binding of ribosomal proteins or other molecules necessary for the successive stages of pre-rRNA maturation. In the nucleolus of the egg cell the factor limiting pre-rRNA maturation is presumably the lack of an appropriate pool of U3 snoRNA, which is required for the processing of the small ribosomal subunit (e.g., Tollervey and Kiss 1997). It is also possible that additional molecules bind to pre-rRNA and inhibit its maturation; in effect, limited access of the 26S rRNA probe to the immature transcript is observed. Studies in mice (Pestov et al. 2001) and humans (Deisenroth and Zhang 2010) have shown that ribosome maturation is coordinated with cell cycle progression. Base on indirect investigations in *Nicotiana tabacum* (Tian et al. 2005) and *Arabidopsis* (Ronceret et al. 2008), the egg cell is believed to be arrested at the G1/S transition (Berger et al. 2008). The question of whether these two processes, inhibition of pre-rRNA maturation and arrest of the cell cycle, are coordinated in the egg cell requires further study.

The results obtained in *H. orientalis* indicate that the maternal pre-rRNAs accumulated in the nucleolus mature soon after fertilization, as 5S rRNA, which occurs at the site of assembly of ribosomal subunits, was observed in almost the whole volume of the nucleolus. After fertilization, rRNA synthesis and maturation is resumed, including synthesis from the paternal rDNA. The *H. orientalis* sperm cells do not have a nucleolus (Zienkiewicz et al. 2011), and shortly after fertilization two nucleoli that contain a high level of pre-rRNA become visible in the zygote. The appearance of these nucleoli is accompanied by the synthesis of a new pool of U3 snoRNA, which accumulates within both zygote nucleoli. The post-fertilization change in the localization pattern of the analysed rRNAs and U3 snoRNA reflects the high activity of the nucleolus. In both nucleoli of the zygote, the 26S rRNA probe detected the transcript almost throughout their entire volume, including areas in which pre-rRNA and U3 snoRNA were present. In addition the increase in 26S rRNA levels in the nucleoplasm may indicate the intense transport of these molecules to the cytoplasm. The intensification of the process of ribosome assembly in zygote nucleoli was previously observed by genetic analysis in *Zea*. The presence of the transcripts of three ribosomal proteins has already been observed in the egg cell; however, the levels of two of them (*ZmrpL39* and *ZmrpS21A*) increase strongly after fertilization (Dresselhaus et al. 1999a). The biogenesis of a new ribosome pool is presumably linked to an intensification of translation, which takes place in the fertilized egg cell.

The resumption of rRNA transcription in the *H. orientalis* zygote indicates that a different mechanism regulates RNA Pol I activity in plants than in animals. In animals, eggs store rRNA partially matured or in the form of a precursor. Only fertilization initiates the formation of functional rRNA, but it does not induce the activation of RNA Pol I (Davidson 1986; Verheggen et al. 1998). The resumption of rRNA synthesis takes place only at the stage of the young embryo (Laurincik et al. 2003; Zatsepina et al. 2003; Svarcova et al. 2008).

In the mature central cell which has a large pool of cytoplasmic rRNA, the nucleolus still seems to be active. In this region, distinctly higher levels of U3 snoRNA and rRNA transcripts were present, both immature ones and those visualized by probes for the 26S and 5S RNAs. During this period, intensive transport of newly formed ribosomal subunits to the cytoplasm also occurs, which is indicated by a high rRNA signal at the boundary between the nucleus and the cytoplasm. The process of ribosome accumulation in the cytoplasm of the maturing central cell was previously observed in *Zea* (Dow and Mascarenhas 1991a, b). Our investigations indicate that, in *H. orientalis*, ribosome maturation also takes place during the progamic phase. In this period, the nucleolus of the central cell still contained a relatively high U3 snoRNA pool, and the 26S rRNA level in the perinuclear cytoplasm was distinctly higher than that before pollination. In turn, a decrease in the rRNA pool detected by the 26S probe, while a high level of pre-rRNA was still present, may indicate an increasing inhibition of rRNA maturation. The nucleolus of the central cell probably also stores immature transcripts that are processed after fertilization.

Shortly after fertilization, three oval structures containing pre-rRNA were visible in the nucleolus of the primary endosperm cell of *H. orientalis*, which indicated that the activity of the paternal rDNA had resumed. In this cell, the synthesis of a new pool of U3 snoRNA also took place, which was observed in the nucleoplasm and in the nucleolus. During syncytium formation in endosperm nuclei only one large nucleolus was present, which contained a large pool of pre-rRNA and 26S rRNA. Thus, in *H. orientalis*, the fertilization of the central cell initiates a strong activation of transcription and the maturation of maternal and paternal rRNA.

In conclusion, our data indicate that, in the egg and central cells, whose activity is silenced in the mature embryo sac, nucleolar activity is directed at the accumulation of rRNPs in the cytoplasm and of immature transcripts in the nucleolus. In both cells, fertilization initiates the maturation of maternal pre-rRNA and the resumption of the expression of zygotic rDNA. In contrast, in synergids and antipodal cells, which have a somatic function, the nucleolar activity is correlated with the metabolic activity of these cells, which changes in successive stages of embryo sac development.

## Electronic supplementary material

Below is the link to the electronic supplementary material.

Fig. 1 Control reactions. **a** Negative FISH control: FISH without labelled probe, the somatic cells are completely devoid of the fluorescence. **b**–**e** Positive FISH controls using the probes as indicated. 26S rRNA and 5S rRNA labelling is present in the cytoplasm and the nucleoli of the somatic cells. Signals of ITS1 pre-rRNA and U3 snoRNA are observed in the nucleoli of these cells; blue, DAPI staining. Bar = 10 μm.

Supplementary material 1 (TIFF 1871 kb)

## Abbreviations

BMM: Butyl methyl methacrylate
DAPI: 4′,6-Diamidino-2-phenylindole
FISH: Fluorescence in situ hybridization
PBS: Phosphate buffered saline
U3 snoRNA: U3 small nucleolar RNA
