# Supplementary figures and images for: Ribosomal RNA of Hyacinthus orientalis L. female gametophyte cells before and after fertilization
Source: Planta. 2012 Mar 8;236(1):171–84. doi: 10.1007/s00425-012-1618-x (PMC3382635; doi:10.1007/s00425-012-1618-x)

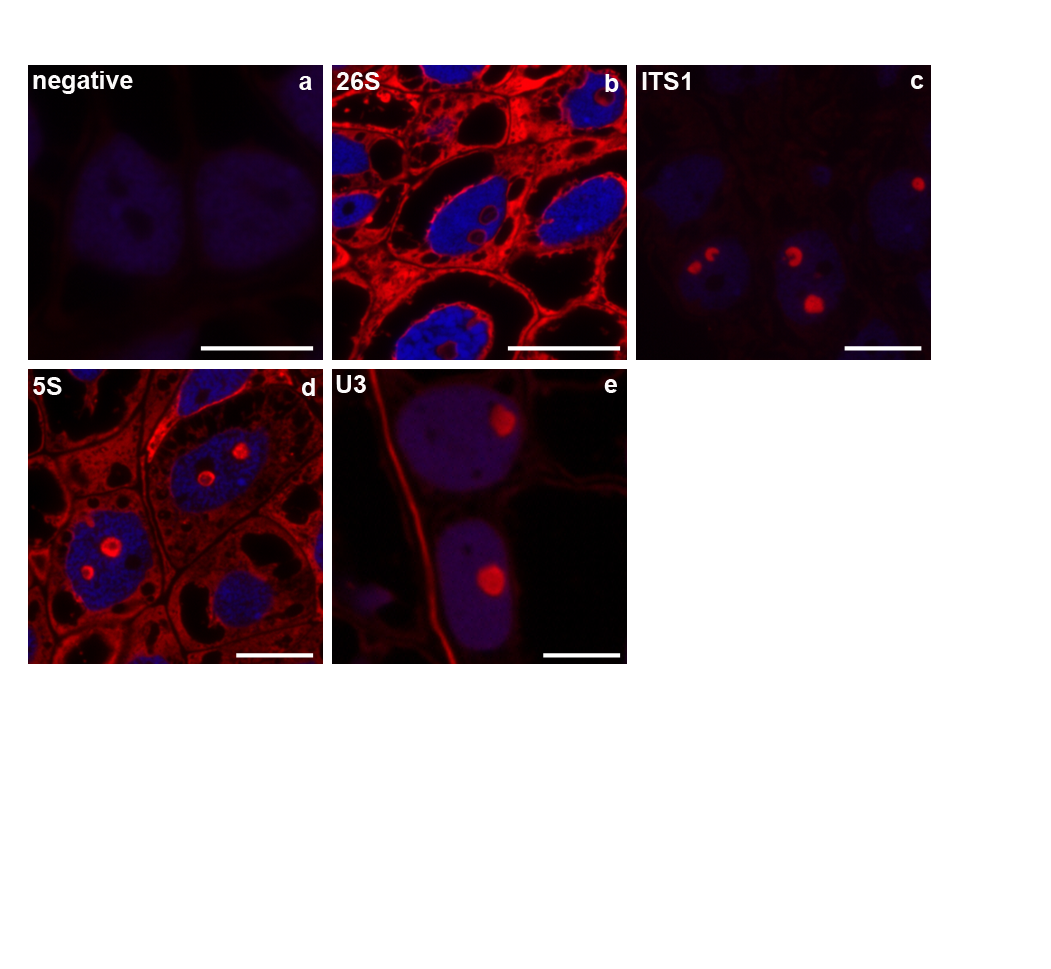

Supplement: Supplementary file 1 — Supplementary material 1 (TIFF 1871 kb) [file 425_2012_1618_MOESM1_ESM.tif]
